# Distal humerus physeal fractures in children under 3 years: a systematic review and quantitative descriptive analysis

**DOI:** 10.1007/s00256-026-05213-3

**Published:** 2026-04-21

**Authors:** Jie C. Nguyen, Carlos Yaya-Quezada, Samuel S. Hailu, Julian Forero-Millan, Benjamin H. Taragin

**Affiliations:** 1 Department of Radiology, Section of Musculoskeletal Imaging, Children’s Hospital of Philadelphia, 3401 Civic Center Blvd, Philadelphia, PA 19104 USA; 2 Perelman School of Medicine, University of Pennsylvania, Philadelphia, PA USA

**Keywords:** Children, Distal humerus, Elbow, Growth plate, Imaging, MRI, Pediatric, Physeal injury, Radiographs, Salter-Harris fracture, Systematic review, Transphyseal separation, Ultrasound

## Abstract

**Objective:**

To conduct a systematic review on the imaging diagnosis and modality-specific characterization of distal humerus physeal fractures among children under 3 years of age.

**Methods:**

A literature search was performed to identify peer-reviewed publications that included imaging descriptions on distal humerus physeal fractures in children < 3 years. Study quality and risk of bias were evaluated. Demographic information, mechanisms of injury, imaging workup and findings, radiographic diagnosis, diagnostic imaging modality, treatment, and outcome were collected and summarized using descriptive statistics.

**Results:**

Thirty-seven studies (published, 1963–2023) included 24 case reports and 13 case series, yielding 101 children (102 elbows), who underwent 175 imaging examinations (102 initial, 7 repeat radiographs; 48 initial, 1 repeat ultrasounds; and 17 MRI examinations). Excluding 3 elbows that underwent concurrent ultrasound, physeal fracture was suspected on radiographs in 55.6% (55/99) and the most common misdiagnosis was joint subluxation-dislocation (53.8%, 21/39). Physeal fractures were identified on ultrasound and/or MRI in 97.9% (47/48) and 94.1% (16/17) of the examinations, respectively. The most reported radiographic finding was posteromedial forearm malalignment (68.6%, 48/70). Most (63.5%, 61/96) elbows were treated with immobilization and residual findings were reported in 44.8% (43/96) of the elbows with the most common findings including cubitus varus (39.5%, 17/43), residual deformity (27.9%, 12/43), and limited range of motion (25.6%, 11/43).

**Conclusion:**

Radiographs were the most often used initial imaging to screen for distal humerus physeal fractures in children under 3 years, whereas ultrasound and MRI were reserved for workup and confirmation. Nearly half of these elbows had complications.

**Supplementary Information:**

The online version contains supplementary material available at 10.1007/s00256-026-05213-3.

## Introduction

Injury involving the distal humerus epiphyseal growth plate, physeal fractures (also known as transphyseal or epiphyseal fracture, separation, or fracture-separation, and Salter-Harris fractures) preferentially affect the extremely immature elbows of infants and young toddlers. While these fractures are uncommon, delayed or missed diagnoses can increase the risk for secondary injuries and future morbidity, which can include cubitus deformity, limited range of motion, and premature osteoarthritis [1–3]. Confident diagnosis is often challenging due to non-specific symptoms, incomplete history, and underdeveloped anatomic landmarks [4, 5]. A retrospective multicenter study that included 79 surgically-treated children with distal humerus physeal fractures found that over half were initially misdiagnosed and clinicians outperformed radiologists in diagnosing these injuries [3]. This highlights the current unmet need to critically examine the frequency of reported suspicion and/or visualization of these injuries on various imaging modalities and to identify imaging findings that can improve the screening and diagnosis of these injuries.

Based on the latest American College of Radiology (ACR) appropriateness recommendations, radiographs remain the recommended first screening imaging modality to evaluate patients presenting with acute elbow pain [6]. However, in contrast to older children and adults, the abundance of unossified growth cartilage in these extremely immature elbows hinders the use of classic alignment methods and diagnostic criteria. Specifically, the capitellar ossification, vital for determining radiocapitellar alignment and alignment along the anterior humeral line, is not visible until 1–2 years of age; and when this ossification first appears, its small size makes radiographic determination of elbow alignment challenging and, less reliable [7–9]. While ultrasound and magnetic resonance imaging (MRI) can help confirm and further characterize suspected physeal injury, an improved understanding on the frequency of reported suspicion of these injuries on radiographs, including patterns of findings and common misdiagnoses, is important for the formulation of future guidelines to improve patient triage and reduce diagnostic delays. To date, there are a number of case reports and few case series within the published literature, but no prior study has systematically examined the overall sequence of imaging use, or identified modality-specific imaging findings and pitfalls. Thus, the purpose of the current study was to conduct a systematic review on the imaging diagnosis and modality-specific characterization of distal humerus physeal fractures among children under 3 years of age. We hypothesize that radiographs are the most frequently used imaging modality to screen with ultrasound and MRI reserved for additional workup of these elbows. Moreover, we hypothesize that radiographic findings are often subtle and less specific, due to the lack of mature anatomic landmarks, but the direction of malalignment overlaps with those delineated with ultrasound and MRI.

## Methods

Current systematic review adhered to the Preferred Reporting Items for Systematic Reviews and Meta-Analyses of Diagnostic Test Accuracy Studies statement (ie, PRISMA 2020) guidelines [10] and was registered with the PROSPERO International Prospective Register of Systematic Reviews (no. CRD420251178900).

### Study identification & quality assessment

**Fig. 1:**
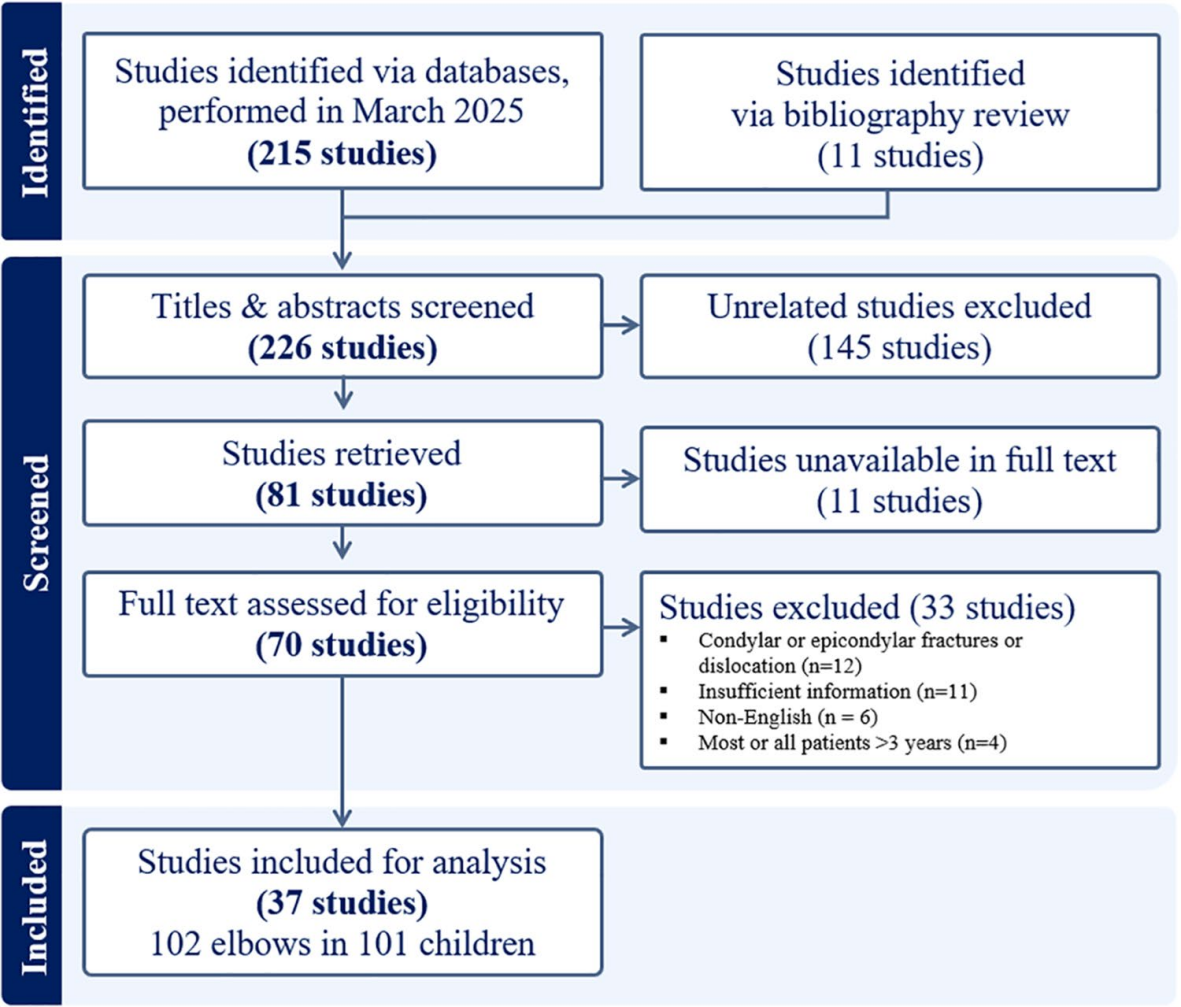
PRISMA (Preferred Reporting Items for Systematic Reviews and Meta-analyses) flow diagram. PubMed, Scopus, and Embase databases were used. Database searches were performed using a combination of root words that include “distal humerus,” “fracture,” “separation,” and “physeal,” yielding a total of 215 unique studies.

In March 2025, a literature search was performed using PubMed, Scopus, and Embase databases to identify peer-reviewed publications that included imaging description on distal humerus physeal fractures in patients under 3 years of age. A combination of Medical Subjects headings (MeSh) and keywords were used, which included “distal humerus,” “fracture,” “separation,” and “physeal,” yielding a total of 215 unique studies (Supplemental Materials). Eligible study designs included peer-reviewed prospective and retrospective primary scientific studies (ie: case reports, case series, cross-sectional, observational, case–control) that included imaging description and findings on distal humerus physeal fracture, and/or clinical and imaging follow-up on fracture healing. Conference abstracts, book chapters, expert opinions, editorials, commentaries, reviews, and systematic reviews were excluded using database-specific filters applied at the time of the search query. Bibliographies of retrieved studies were manually screened to identify any additional relevant studies for inclusion (*n* = 11). Studies that did not report on the use of imaging (*n* = 145) and those without available full text (*n* = 11) were excluded. All studies with potentially relevant titles and abstracts underwent full text review (*n* = 70). Additional studies that reported on condylar or epicondylar fractures or elbow dislocation (*n* = 12), contained insufficient information (*n* = 11), written in non-English language (*n* = 6), or included most or all patients > 3 years (*n* = 4) were excluded (Fig. 1).

The Covidence software (Veritas Health Innovation, used in 2023) was used for the initial screening of titles and abstracts, and the subsequent assessment of full text studies to determine eligibility for inclusion. A pediatric radiologist (SSH with over 4 years of post-training clinical experience) and a medical graduate (CY with 2 years of musculoskeletal research experience) independently reviewed all studies. Disagreements were reviewed by another pediatric radiologist (BHT with 22 years of post-training clinical experience), who made the final determination for inclusion. No automated or semiautomated software was used to complete any of these tasks.

The modified versions of JBI (Joanna Briggs Institute) Critical Appraisal Checklists for Case Reports [11] and Case Series [12] were used to assess study quality and risk of bias. For case reports, 7 domains were used to appraise each study, which included (i) clarity of demographic information, (ii) a clearly outlined, chronological clinical history, (iii) an adequate description of the patient’s condition at presentation, (iv) a transparent account of diagnostic tests, assessment methods, and corresponding results, (v) a clear description of the post-diagnosis clinical course, (vi) identification and discussion of any adverse or unanticipated events, and (vii) the inclusion of explicit takeaway lessons. For case series, 8 domains were used to appraise each study, which included (i) the presence of clear inclusion criteria for participants, (ii) standardized and reliable measurement of the condition for all participants, (iii) use of valid methods to identify the condition, (iv) consecutive inclusion of eligible participants, (v) complete inclusion of all eligible participants within the specified timeframe, (vi) clear reporting of participant demographics, (vii) clear reporting of clinical information for each participant, and (viii) clear reporting of outcomes or follow-up results. Low risk of bias was defined as having an overall score > 70%. All assessments were performed independently by a pediatric radiologist (SSH) and the medical graduate (CY). Disagreements were resolved through a separate consensus review.

### Data extraction

For each study, demographic information (age, sex, laterality of injury), mechanisms of injury, imaging workup and findings, radiographic diagnosis (or misdiagnosis), diagnostic imaging modality, treatment, and outcome were collected. Imaging workup included the sequential use of imaging modalities. For completeness, additional follow-up information, include the use of imaging, imaging modality, and imaging findings were also recorded. Initial data collection was performed by the pediatric radiologist (SSH) and the medical graduate (CY) under the supervision of a pediatric musculoskeletal radiologist (JCN with over 12 years of post-training clinical experience), who also double-checked all recorded data used for analysis.

### Statistical analysis

Statistical analysis was performed using R (version 4.3.2; R Foundation for Statistical Computing, Vienna, Austria) and R Studio (version 2023.12.1; Posit Software, PBC, Boston, MA). Descriptive statistics were used with categoric variables presented as counts and percentages, and continuous variable as mean ± standard deviation (SD) if normally distributed and median with interquartile range (IQR) if not normally distributed. Kolmogorov–Smirnov tests were used to assess the normality of continuous variables.

Cohen kappa (κ) was used to calculate interreader agreement for assessment of study quality and risk of bias, categorized as slight (≤ 0.20), fair (0.21–0.40), moderate (0.41–0.60), substantial (0.61–0.80), almost perfect (0.81–0.99), and perfect agreement (1) [13]. Median age at diagnosis was compared between birth-related injury and non-accidental trauma using the Mann–Whitney U test. Statistical significance was defined as a *P*-value < 0.05.

## Results

### Study identification & quality assessment

From the 226 unique studies, 37 (16.4%) met the inclusion and exclusion criteria for inclusion. Interreader agreement was substantial (κ = 0.61) for title and abstract screening and near perfect (κ = 0.89) for full-text screening. A PRISMA flow diagram summarized the study selection process (Fig. 1). These 37 studies were published between 1963–2023, which included 24 case reports and 13 case series. All 24 case reports were apprised as low risk of bias with perfect interreader agreement (κ = 1.00).  For the 13 case series, 76.9% (10/13) were apprised as low and and 23.1% (3/13) [14–16] as moderate risk of bias with substantial (κ = 0.76) interreader agreement (Supplemental Materials).

### Demographics & clinical findings

Twenty-four case reports (Table 1) included 24 children (25 elbows), and 13 case series (Table 2) included 77 children (77 elbows), which yielded a total of 101 children (51 boys, 33 girls, and 17 unknown sex) and 102 elbows (46 right, 31 left, and 25 unknown laterality). An 8-day-old child (unknown sex) sustained bilateral distal humerus physeal fractures [17]. The mechanism of injury was reported for 96 elbows, which included birth-related injuries (53.9%, 55/96), non-accidental trauma (24.5%, 25/96), falls (12.7%, 13/96), injury from sibling (2.0%, 2/96), and arm traction (1.0%, 1/96). The median age at presentation was significantly lower for elbows with injuries from birth-related than non-accidental trauma (2 days, IQR: 1–6, vs. 165 days, IQR: 99–405; P < 0.01). Symptoms at presentation was reported for 72 elbows, which included one or a combination of elbow swelling (93.1%, 67/72), limited range of motion (59.7%, 43/72), pain, tenderness, or discomfort with movement (52.8%, 38/72), palsy or (pseudo)paralysis (19.4%, 14/72), crepitus (13.9%, 10/72), and non-specified deformity (6.9%, 5/72).

**Table 1:** Radiographic findings and diagnostic imaging modality among case reports with children under 3 years of age with distal humerus physeal fractures

Authors	Age (Sex)	MOI	L/R	Fracture on CR	Alignment on CR	Reported Diagnosis on CR	Additional imaging	Diagnostic Modality
Arrigoni, Sini & Origo, 2022 [26]	3-DO (F)	B	R	-	Loss of joint congruence, forearm posterior to humerus	-*	US: Epiphyseal separation (SH I)	US
Beckmann & Crawford, 2017 [38]	2-DO (F)	B	L	None	1 st CR: Forearm PM to humerus 2nd CR (same day): Forearm PM to humerus	1st: Dislocation* 2nd: SH I	US: Physeal “step-off” (SH I), epiphysis PM displaced	Repeat CR
Brown & Eustace, 1997 [19]	3-DO (F)	B	R	Medial humerus	Decreased distance between anterior humeral line & radius	Fracture, metaphyseal (NOS)	US: Discontinuous cortex, distal humerus lateral to shaft MRI: Physeal fracture, epiphysis posterior displaced	US + MRI
Chand, 1974 [24]	7-MO (F)	NAT	L	Distal humerus, NOS	1 st CR: Epiphysis posterior displaced, radiocapitellar aligned 2nd CR (+ 12 days): Forearm & epiphysis PM to humerus	1st: NR 2nd: Dislocation*	Arth: Epiphysis PM displaced	Arth
Cohen et al*.*, 2006 [27]	14-DO (F)	B	R	-	Forearm medial to humerus	-*	US: Physeal separation, epiphysis PM displaced, periosteal elevation	US
Costa et al*.*, 2001 [39]	19-DO (M)	NR	R	None	Elbow rotation with lateral forearm and AP humerus	Fracture (NOS) versus dislocation	MRI: Epiphysis posterior displaced (angulated, & rotated), periosteal stripping & maintained joints	MRI
Dias, Lamont, & Jones, 1988 [28]	0-DO (F)	B	R	-	Forearm PM to humerus	Dislocation*	Arth: Physeal separation, epiphysis PM displaced US: Confirmed, epiphysis posterior displaced	US
Downs & Wirth, 1982 [29]	1-DO -	B	R	None	1 st CR: Forearm posterior to humerus 2nd CR (after transfer): Forearm PM to humerus	1st: Dislocation* 2nd: physeal fracture	-	Repeat CR
Hansen et al., 2008 [30]	5-MO (F)	NAT	R	None	Forearm medial to humerus	Dislocation*	US: Separation, epiphysis medial displaced, subperiosteal hematoma; MRI: Confirmed, NOS	US + MRI
Hansen, Barnes & Tullos, 1982 [23]	11-MO (M)	NR	R	Lateral humerus	No “gross displacement”	SH II or SH IV	Arth: Epiphyseal separation (SH II)	CR
Kamaci, Danisman & Marangoz, 2014 [40]	2-DO (M)	B	R	-	Forearm medial to humerus with PM humeroulnar joint	-*	Arth: Epiphysis PM displaced	Arth
Lin, Liu & Zhang, 2016 [41]	5-DO (F)	B	R	-	Forearm PM to humerus	-*	MRI: Epiphysis PM displaced, maintained joints	MRI
Malik et al*.*, 2015 [18]	0-DO (M)	B	R	-	1 st CR: NR 2nd CR (next day): Forearm PM to humerus 3rd CR (+ 14 days): SH II	1st: NR 2nd: Dislocation 3rd: SH II	US: Soft tissue swelling US (+ 14 days): Epiphysis PM displaced (SH II), healing changes	Repeat CR + US
Merten, Kirks, & Ruderman, 1981 [20]	6-MO (M)	NAT	R	Medial humerus	Forearm medial to humerus	Fracture, NOS	Arth: Fracture-separation, NOS	Arth
Moucha & Mason, 2003 [42]	4-MO (F)	NAT	L	None	Forearm PM to humerus	Physeal fracture & separation	MRI: Fracture-separation PM displaced	CR
Navallas et al*.*, 2013 [43]	0-DO (F)	B	R	None	Forearm PM to humerus	Dislocation*	US: Epiphysis PM displaced, maintained joints	US
Rose, Dixon & Dullon, 2002 [44]	0-DO -	B	R	-	1 st & repeat CR (NOS): Forearm PM to humerus	Dislocation*	Arthrotomy: distal humerus devoid of cartilage; US: Epiphysis PM displaced	US
Sawant et al*.*, 2002 [45]	0-DO (M)	B	R	-	Forearm medial to humerus (Fig. 1)	Dislocation*	MRI: Fracture-separation, epiphysis posterior displaced, maintained joints	MRI
Siddiqui [17]et al*.*, 2023	8-DO -	NAT	L + R	-	Forearm PM to humerus	Physeal separation	US (at presentation): Physis “step-off,” epiphysis PM displaced	CR + US
Soyuncu et al*.*, 2009 [46]	2-DO (M)	B	R	None	1 st CR & 2nd CR (+ 3 days): Forearm PM to humerus	Dislocation*	US: “Changed relationship” between epiphysis & metaphysis MRI: Fracture-separation, NOS	US + MRI
Tharakan et al*.*, 2016 [47]	1-DO (M)	B	R	-	Forearm PM to humerus	“Elbow injury” NOS	US: Epiphysis posterior displaced, maintained joints	US
Triplet, Samora & Samora, 2018 [21]	4-DO (M)	B	L	Medial humerus	Forearm PM to humerus	Dislocation*	-	Surgery
Varghese et al*.*, 2017 [48]	0-DO (F)	B	L	-	Forearm PM to humerus	Dislocation*	US: Physeal fracture, epiphysis PM displaced, maintained joint	US
Wang et al*.*, 2009 [31]	1-DO (M)	B	R	None	“Nonspecific angulation”	Subluxation*	US: Epiphysis PM displaced MRI: Confirmed, NOS	US + MRI

*Arth* arthrogram, *B* birth-related injury, *CR* computed radiography, *DO* day-old, *F* female, *M* male, *MO* month-old, *MOI* mechanism of injury, *MRI* magnetic resonance imaging, *NAT* non-accidental trauma, *NOS* not otherwise specified, *NR* not reported, *PM* posteromedial, *SH* Salter-Harris, *US* ultrasound. *Misdiagnoses

**Table 2:** Radiographic findings and diagnostic imaging modality from studies with 2 or more children under 3 years of age with distal humerus physeal fractures

Authors	Age (Sex)	MOI	L/R	Fracture on CR	Alignment on CR	Reported Diagnosis on CR	Additional imaging	Diagnostic Modality
Davidson et al*.*, 1994 [25]	4-DO 2-MO 17-MO (3 M)	1 B 2 NAT	NR	-	-	Dislocation (4-DO)* Transphyseal fracture (2-MO) Fracture-dislocation (17-MO)	US: Transphyseal fracture × 3	2 CR 1 US
Fette & Mayr, 2012 [34]	2-DO 4-MO (2F)	1 B 1 NAT	2L	- (2-DO) None (4-MO)	-	Dislocation (2-DO)* “No fracture” (4-MO)*	US (2-DO): Fracture-separation, posterior epiphysis (MRI: inconclusive) US (4-MO): Separated, variable positioned epiphysis (MRI: confirmed, NOS)	2 US
Galeotti et al*.*, 2023 [49]	0 to 9-DO (7 M:3F)	10 B	5L 5R	-	-	Transphyseal fracture (suspected) (all)	US (2, 2, 3, 6, 6, 9, 9-DO); US + MRI (3, 8-DO): confirmed, PM epiphysis NA (0-DO)	10 CR
Gigante et al*.*, 2017 [33]	0 to 3-DO (5 M)	5 B	1L 4R	-	-	Fracture-separation (0, 1, 2, 3-DO) Dislocation (2-DO)*	US (2-DO): PM Epiphysis US (0-DO): NOS NA (1, 2, 3-DO)	4 CR 1 US
Gilbert & Conklin, 2007 [2]	13-DO to 23-MO (7NR)	3 Falls 2 NAT 1 B 1 Traction	NR	Posterior humerus (15-MO) Non-transphyseal (10, 14, 20-MO) None (13-DO, 6, 23-MO)	Forearm PM to humerus (15-MO) - (others)	Non-physeal fractures: Lateral epicondyle (10-MO), medial condyle (14-MO), supracondylar (20-MO)* Normal (13-DO, 6-MO)* Clinical correlate (23-MO)* “NA” (15-MO)*	-	7 Surgery
Jacobsen, Hansson & Nathorst-Westfelt, 2009 [32]	1 to 30-DO (2 M:4F)	6 B	2L 4R	Fracture, NOS (1, 14, 30-DO) None (2, 9, 12-DO)	Forearm PM to humerus (all)	Fracture, presumed physeal (1-DO, 14-DO, 30-DO) Dislocation (2-DO, 12-DO)* Pain (9-DO)*	US (2-DO, 9-DO): Epiphysis aligned with forearm US + MRI (1-DO): Posterior epiphysis Arth (12-DO): “outline fracture” NA (14, 30-DO)	3 CR 2 US 1 Arth
Kay et al*.*, 2017 [14]	1 to 7-DO (4NR)	4 B	3L 1R	SH II (4-DO) None (1-DO preemie, 1, 7-DO)	Forearm PM to humerus (all)	SH II (4-DO) Dislocation (1-DO preemie, 1-DO, 7-DO)*	US (7-DO): Posterior epiphysis, maintained joints; MRI(1-DO preemie): Separated, posterior epiphysis, maintained joints Arth (4-DO): Transphyseal fracture, posterior epiphysis NA (1-DO)	1 CR 1 US 1 MRI 1 Arth
Nimkin et al*.*, 1995 [15]	3-MO 12-MO 23-MO (2 M:1F)	3 NAT	3L	Medial (3-MO), PM humerus (12-MO) Non-transphyseal (23-MO)	Forearm posterior (3-MO) or PM (12-MO) to humerus - (23-MO)	Physeal fractures (3, 12-MO) Non-physeal fracture (23-MO)	US + MRI (3-MO): Posterior epiphysis (SH II), effusion MRI (12-MO): Posterior epiphysis displaced (SH II) MRI (23-MO): Medial epiphysis (SH III)	2 CR 1 MRI
Oh, Park & Kyung, 2000 [50]	13 to 36-MO (8 M:4F)	12 falls	NR	SH II (all)	Epiphysis PM displacement	SH II (all)	-	12 CR
Paige & Port, 1985 [51]	0-DO 4-MO (1 M:1F)	1 B 1 NAT	2L	-	Forearm medial (0-DO), PM (4-MO) to humerus	Epiphyseal separation (0-DO, 4-MO)	-	2 CR
Rogers & Rockwood, 1973 [22]	7 to 35-MO (1 M:3F)	NR separately	2L 2R	SH II (7-MO) SH II (14-MO) SH II, medial (28-MO) SH I (35-MO)	Forearm PM (7-MO), medial (14, 28, 35-MO) to humerus	Separation with malalignment (all)	-	4 CR
Siffert, 1963 [16]	“Shortly after birth” (3NR)	3 B	NR	None (all)	Foreshortened humerus & forearm, forearm posterior to humerus (all)	“Displaced humeral epiphysis” (all)	-	3 CR
Supakul et al*.*, 2015** [5]	1-DO to 2.3-YO (10 M:6F)	6 B 4 NAT 4 Falls 2 Injuries	11R 5L	Bucket-handle (3.3-MO), Non-transphyseal: (10, 14-DO; 2-YO), and none (all others)	Forearm medial (9 NOS), PM (6 NOS) to humerus (others suboptimal positioning)	Epiphyseal separation (1, 2-DO; 3.3, 8.1-MO; 1.3, 1.3, 1.5-YO); Bucket-handle fracture (3.3-MO) Supracondylar fracture (10, 14-DO; 2-YO); Dislocation (7-DO; 7, 10-MO); Normal (4-DO, 2.3-YO)*	US (2, 4, 7, 10, 14-DO; 3.3, 3.3, 7, 8.1-MO; 1.3, 1.5, 2-YO): Transphyseal fracture, displaced NOS, no dislocation NA (1-DO, 10-MO, 1.3, 2.3-YO)	8 CR 8 US

*Arth* arthrogram, *B* birth-related injury, *CR* computed radiography, *DO* day-old, *F* female, *M* male, *MO* month-old, *MOI* mechanism of injury, *MRI* magnetic resonance imaging, *NAT* non-accidental trauma, *NOS* not otherwise specified, *NR* not reported, *PM* posteromedial, *SH* Salter-Harris, *US* ultrasound, *YO* year-old. *Misdiagnoses, ** Radiographic information from the initial clinical data was extracted.

### Imaging & findings

A total of 175 imaging examinations were performed on 102 elbows (median: 2 examinations per elbow, IQR: 1–2 range, 1–5 examinations per elbow), which included 102 initial and 7 repeat radiographs, 48 initial and 1 repeat ultrasounds, and 17 MRI examinations (Tables 1 and 2). Because the included studies were case reports and case series without controls or age-matched non-fracture comparisons, imaging findings are reported descriptively, and diagnostic accuracy metrics such as sensitivity and specificity could not be determined.

**Fig. 2:**
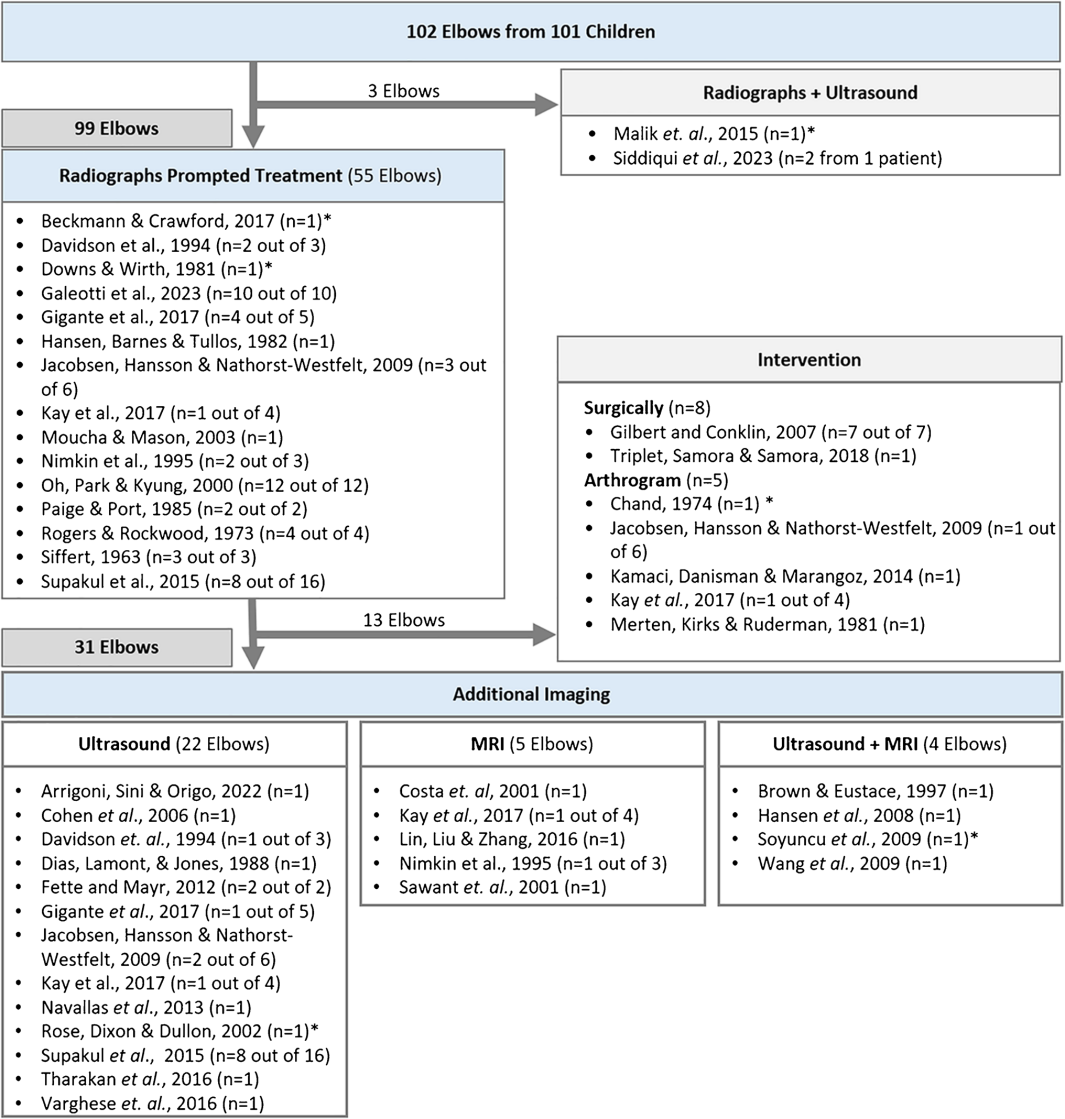
Imaging workup with elbows subdivided based on the diagnostic imaging modality or modalities. Additional ultrasound in 20 elbows included 9 elbows in Galeotti et al*.* [49], 4 elbows in Supakul et al*.* [5], 2 elbows in Davidson et al*.* [25], and 1 elbow each in Beckmann & Crawford[38]; Gigante et al*.* [33]; Jacobsen et al*.* [32]; Malik et al*.* [18]; Nimkin et al*.* [15]. And additional MRI in 8 elbows included 2 elbows each in Fette & Mayr [34], Galeotti et al. [49], Nimkin et al. [15] and 1 elbow each in Jacobsen, Hansson & Nathorst-Westfelt [32], and Moucha & Mason [42]. *Repeat radiographs (*n* = 7) Beckmann & Crawford [38] (same day), Chand [24] (12 days later), Downs & Wirth [29] (after transfer), Malik et al*.*, [18] (next day & 14 days later), Rose, Dixon & Dullon [44] (not otherwise specified), and Soyuncu et al*.* [46] (3 days later)

All elbows underwent radiographic evaluation. Excluding 3 elbows in 2 patients that underwent concurrent ultrasound [17, 18], physeal fractures were suspected on radiographs in 55.6% (55/99) of the elbows. Excluding 5 elbows without radiographic diagnoses, among the remaining 39 elbows with miss diagnoses, subluxation-dislocation was the most common misdiagnosis (53.8%, 21/39), followed by non-physeal or non-specified fractures (25.6%, 10/39), normal elbow or no fracture (12.8%, 5/39), and non-specific diagnoses (7.7%, 3/39 including “clinical correlation,” “injury,” or “pain”). Figure 2 outlines the imaging workup with elbows subdivided based on the diagnostic imaging modality/modalities. Alignment of the proximal forearm relative to the distal humerus was the most frequently reported radiographic finding, described in 70 elbows, which included 68.6% (48/70) with posteromedial, 24.3% (17/70) with medial, and 7.1% (5/70) with posterior malalignments. Distal humerus fracture or the presence of a fracture fragment was an uncommon finding, reported in 10 elbows, located along the medial distal humerus in 50% (5/10), followed by unspecified in 20% (2/10), and 10% (1/10) each along the posteromedial, lateral, posterior distal humerus [2, 5, 15, 19–24]. The presence of the capitellar ossification was rarely reported among the case reports [24] and inconsistently described among the case series [2, 5, 15, 22, 25]. Similarly, comparison radiographs of the contralateral asymptomatic elbow were uncommonly obtained among the case reports [19, 24, 26–31] and not consistently described among the case series [2, 16, 22, 32].

A total of 49 ultrasound examinations were performed on 48 elbows. One elbow was diagnosed after repeat ultrasound (with repeat radiographs) [18]. Excluding this initially non-diagnostic ultrasound [18], physeal fracture was identified in 97.9% (46/47) of the sonographic examinations. The remaining sonographic examination (2.1%, 1/47) lacked imaging detail or diagnosis [33]. Epiphyseal displacement relative to the humeral metaphysis was described in 28 elbows, which included 67.9% (19/28) with posteromedial, 21.4% (6/28) with posterior, 3.6% (1/28) with medial, and 3.6% (1/28) with lateral displacements. A total of 17 MRI examinations were performed on 17 elbows and physeal fracture was identified in 94.1% (16/17). One MRI examination was reported as “inconclusive” [34]. Epiphyseal displacement was described in 12 elbows, which included 58.3% (7/12) with posterior, 33.3% (4/12) with posteromedial, and 8.3% (1/12) with medial displacements. Overall, additional ultrasound and MRI examinations contributed to the final diagnosis of distal humerus physeal fractures in 31.3% (31/99: 22 ultrasound, 5 MRI, and 4 both US and MRI) of the elbows.

### Treatment and outcome

Treatment and follow-up information were reported in 94.1% (96/102) of the elbows. Treatment included immobilization with or without reduction or traction in 63.5% (61/96) of the elbows, closed reduction with percutaneous pinning (CRPP) in 27.1% (26/96), and open reduction in 9.4% (9/96). Imaging examinations were obtained at follow-up in 76.0% (73/96) of the elbows, which included radiographs in 94.5% (69/73), and both radiographs and ultrasound in 5.5% (4/73). Residual clinical and imaging findings were reported in 44.8% (43/96) of the elbows, which included cubitus varus-valgus alignment (varus, 39.5%, 17/43; valgus, 2.3%, 1/43), residual deformity (27.9%, 12/43), limited range of motion (25.6%, 11/43), pain or paresthesia (4.7%, 2/43) (Tables 3 and 4).

**Table 3:** Treatment and outcome information from case reports with children under 3 years of age with distal humerus physeal fractures

Study #	Age (Sex)	Treatment	Follow-Up Duration	Clinical Findings	Imaging & Outcomes
Arrigoni, Sini & Origo, 2022 [26]	3-DO (F)	CRPP	6 months	Symmetric alignment, restored motion	CR (3 weeks): Callus CR (6 months): Healed
Beckmann & Crawford, 2017 [38]	2-DO (F)	CRPP	2 months	Full ROM	CR: Normal alignment
Brown & Eustace, 1997 [19]	3-DO (F)	Immobilization (cast)	3 weeks	Normal ROM & normal alignment	-
Chand, 1974 [24]	7-MO (F)	Immobilization (cast)	18 weeks	Normal motion & no deformity	CR: Return to normal anatomy
Cohen et al*.*, 2006 [27]	14-DO (F)	Immobilization (splint)	6 months	“Uneventful recovery”	CR: “Uneventful recovery”
Costa et al*.*, 2001 [39]	19-DO (M)	Immobilization (traction)	1 month	Normal carrying angle, full ROM	-
Dias, Lamont, & Jones, 1988 [28]	0-DO (F)	Immobilization (collar & cuff)	4 weeks	Full ROM, no deformity	CR (8 days): Callus formation
Downs & Wirth, 1982 [29]	1-DO -	Immobilization (sling/swathe)	6 months	Normal carrying angle, lacked 12° of extension	CR: Remodeling
Hansen et al., 2008 [30]	5-MO (F)	OR	1.5 years	No motion restriction	CR: 10° cubitus varus, congruent humero-ulnar alignment
Hansen, Barnes & Tullos, 1982 [23]	11-MO (M)	Immobilization (splint)	6 months	Full ROM, no deformity	CR (3 weeks): Healing CR (6 months): Normal
Kamaci, Danisman & Marangoz, 2014 [40]	2-DO (M)	CRPP	6 months	Full ROM	CR (3 weeks): Callus formation CR: (6 months): Fracture union
Lin, Liu & Zhang, 2016 [41]	5-DO (F)	CRPP	2 months	Full ROM, symmetric carrying angle,	CR: Normal alignment
Malik et al*.*, 2015 [18]	0-DO (M)	Immobilization (splint)	2 months	Full ROM, no shortening “Minor” cubitus varus	CR: Remodeled healed fracture
Merten, Kirks, & Ruderman, 1981 [20]	6-MO (M)	NR	-	-	-
Moucha & Mason, 2003 [42]	4-MO (F)	Immobilization (Cast)	10 months	No abnormality	No abnormality
Navallas et al*.*, 2013 [43]	0-DO (F)	Immobilization (cast)	4 months	No motion restriction	US (8 days): Callus CR (4 months): Remodeling, residual carrying angle with 10° varus
Rose, Dixon & Dullon, 2002 [44]	0-DO -	OR	6 months	“Mild” cubitus varus	CR: “Mild” cubitus varus
Sawant et al*.*, 2002 [45]	0-DO (M)	NR	-	-	-
Siddiqui [17]et al*.*, 2023	8-DO -	Immobilization (splint), bilateral	1 year	Right: Cubitus varus Left: Cubitus valgus	CR (3 months): Healing CR (1 year): Healing
Soyuncu et al*.*, 2009 [46]	2-DO (M)	OR	16 months	Full ROM	CR: NOS
Tharakan et al*.*, 2016 [47]	1-DO (M)	CRPP	1 year	No deformity, full ROM	CR (1 week): Healing CR (6 weeks): Remodeling CR (4 months): Remodeling
Triplet, Samora & Samora, 2018 [21]	4-DO (M)	CRPP	4 years	Asymmetric ROM	CR (3 weeks): Callus CR (25 weeks): Callus CR (4 years): Metaphyseal flare, symmetric carrying angle
Varghese et al*.*, 2017 [48]	0-DO (F)	-NR	-	-	-
Wang et al*.*, 2009 [31]	1-DO (M)	CRPP	4 years	Normal ROM, symmetric carrying angle	CR & US (2 weeks): Callus, normal alignment CR & US (2 months): Fracture union CR & US (4 years): Healed

*B* possibly birth-related, *C* capitellum ossification, *CR* computed radiography, *CRPP* closed reduction percutaneous pinning, *DO* day-old, *F* female, *M* male, *NOS* not otherwise specified, *NR* not reported, *OR* open reduction, *ORIF* open reduction internal fixation, *ROM* range of motion, *US* ultrasound

**Table 4:** Treatment and outcome information among studies with 2 or more children under 3 years of age with distal humerus physeal fractures

Study #	Age	Treatment	Follow-Up Duration	Clinical Findings	Imaging & Outcomes
Davidson et al*.*, 1994 [25]	4-DO 2-MO 17-MO (3 M)	3 CRPP	Minimum 3 months, up to 27 months (among all 8 included patients)	-	CR: Reduced & healing
Fette & Mayr, 2012 [34]	2-DO 4-MO (2F)	2 Immobilization (sling)	6 months (2-DO) 8 months (4-MO)	Slight joint restriction (2-DO) Full ROM (4-MO)	CR & US: Healing, joint aligned
Galeotti et al*.*, 2023 [49]	0 to 9-DO (7 M:3F)	5 Immobilization (cast) 5 CRPP	12–120 months	Full ROM & normal alignment (all) 5th finger paresthesia (0-DO) Occasional elbow pain (8-DO)	CR (26–31 days): Healing CR (latest follow-up): No physeal injury, normal radiocapitellar alignment, Baumann & shaft-condylar angles
Gigante et al*.*, 2017 [33]	0 to 3-DO (5 M)	4 Immobilization (cast) 1 CRPP	12–60 months	Full ROM (all) 5° Cubitus varus (0-DO Preemie, at 12 months)	CR (1, 2, 4 weeks, 6 months, 1 year): Healing (all)
Gilbert & Conklin, 2007 [2]	13-DO to 23-MO (7NR)	4 Immobilization 3 CRPP	Variable (up to 12 months)	Limited ROM (1 patient, NOS) Deformity & extension loss (1 patient, NOS) Extension loss, 5° (1 patient, NOS)	-
Jacobsen, Hansson & Nathorst-Westfelt, 2009 [32]	1 to 30-DO (2 M: 4 F)	6 Immobilization (cast)	16–120 months	Extension loss, 5° (1 patient, NOS) “Slightly reduced valgus” (1-DO)	CR (follow-up): Healed (2, 9, 12, 14, 30-DO), abnormal carrying angle (reduced valgus, 1-DO)
Kay et al*.*, 2017 [14]	1 to 7-DO (4NR)	3 Immobilization 1 CRPP	15 days – 13 months	Normal alignment (all)	CR (follow-up): Fracture union, remodeling & normal alignment (all)
Nimkin et al*.*, 1995 [15]	3-MO 12-MO 23-MO (2 M: 1 F)	3 NR	-	-	-
Oh, Park & Kyung, 2000 [50]	13 to 36-MO (8 M: 4 F)	6 CRPP 6 Immobilization (cast)	12–46 months	Normal carrying angle (14, 16, 20, 20, 20-MO) 5° −15° Cubitus varus (13, 13, 15, 18, 20, 22, 36-MO)	-
Paige & Port, 1985 [51]	0-DO 4-MO (1 M: 1 F)	2 Immobilization (splint/mold)	“Long-term” (0-DO) NOS (4-MO)	Full ROM (0-MO)	None (0-DO) CR (Follow-up): Callus & periosteal new bone (4-MO)
Rogers & Rockwood, 1973 [22]	7 to 35-MO (1 M: 3 F)	3 Immobilization (splint) 1 ORIF	13 days (14-MO) “Short-term” (7, 28, 35-MO)	No growth disturbance No limited motion	CR (7–10 days): Periosteal new bone formation (all)
Siffert, 1963 [16]	“Shortly after birth” (3NR)	3 Immobilization (splint/tape)	7–26 months	Lacked 10° terminal extension & 10° cubitus varus (1) Lacked 10° terminal flexion (1) Limitation in flexion–extension by 10–15° (1)	CR (follow-up): Broadening (2) & anterior angulation of distal humeri (1)
Supakul et al*.*, 2015** [5]	1-DO to 2.3-YO (10 M:6F)	11 Immobilization (cast) 5 ORIF	0.5–15 months	Full ROM (10)	CR: Medial position of capitellum (1, 2, 10-DO, 7-MO, 2, 2.3-YO), irregular humeral condyle (2, 4-DO) Varus deformity (1.3, 2-YO)

*B* possibly birth-related, *C* capitellum ossification, *CR* computed radiography, *CRPP* closed reduction percutaneous pinning, *DO* day-old, *F* female, *M* male, *NOS* not otherwise specified, *NR* not reported, *OR* open reduction, *ORIF* open reduction internal fixation, *ROM* range of motion, *US* ultrasound, *YO* year-old

## Discussion

This systematic review with quantitative descriptive analysis of 37 case reports and case series addresses a current unmet need to examine the imaging workup and the use of various imaging modalities to diagnose distal humerus physeal fractures in the extremely immature elbows of infants and young toddlers. Our results confirmed that radiographs were the first imaging modality used to screen for these injuries, but were diagnostic in only slightly over half of the injured elbows; and the most common radiographic misdiagnosis was joint subluxation-dislocation. Ultrasound and MRI were used to confirm some of these physeal fractures, and both modalities allow direct visualization of the site of physeal injury in over 90% of the imaged elbows.

The relative abundance of unossified cartilage in the extremely immature elbow has been postulated to contribute to the limited visualization and often subtle findings on radiographs [3]. Specifically, the capitellar ossification, the first of six secondary ossification centers of the elbow to ossify, is not visible until 1–2 years of age [7]. Its absence in these immature elbows hinders assessment of radiocapitellar alignment and alignment across the anterior humeral line, both of which help distinguish between distal humerus physeal fracture and elbow joint subluxation-dislocation. This may explain the observation that joint subluxation-dislocation accounted for over half of the radiographic misdiagnoses. Specifically, alignment between the proximal forearm and distal humerus was described in over two-thirds of the injured elbows, which in the absence of the capitellar ossification, hinders the distinction between physeal fracture (occurring with mainatained radiocapitellar alignment) and elbow dislocation (occurring with abnormal radiocapitellar alignment). However, it is important to recognize that in the skeletally immature, the physis is the physiologic “weak link” and is prone to torsional and tractional forces that can produce complete osteochondral disruption [4, 22]. Moreover, dislocations of the elbow joint is uncommon [35] and the peak incidence of elbow dislocations is typically during the second decade of life [36]. Among toddlers with relatively small, newly formed capitellar ossifications, it is important to also recognize that the anterior humeral line can transect the anterior third of the ossification (as opposed to the typical middle third) in up to 40% of elbows in children under 4 years of age, which should not be mistaken for physeal fracture with posterior displacement [8, 9].

Ultrasound and MRI allowed direct visualization of the unossified cartilage, and both modalities frequently allowed identification of physeal fractures, but neither reached 100%. On ultrasound, excluding the single examination that lacked imaging detail and diagnosis [33], one ultrasound examination was diagnostic only at follow-up [18] and the initial non-diagnostic ultrasound examination only reported soft tissue swelling. These examples emphasize the operator dependence in the assessment and reporting of these injuries, a well-recognized disadvantage with ultrasound. On MRI, one examination was “inconclusive,” which was performed after sonographic diagnosis of distal humerus epiphyseal fracture with posterior displacement [34]. This example highlights the need for better defined guidelines for the imaging workup of these patients. Our results showed that although 49 ultrasound and 17 MRI examinations were performed, less than half directly contributed to the initial diagnosis of distal humerus physeal fracture that prompted treatment.

Although radiographs do not allow the direct visualization of the cartilaginous epiphysis of the distal humerus, the preferential posteromedial malalignment of the forearm overlaps with the frequent sonographic and MRI observation of posteromedial and posterior displacement across the fracture. Thus, in these extremely immature elbows, apparent malalignment of the forearm relative to the distal humerus, particularly when it is asymmetric compared to the contralateral asymptomatic elbow should alert the reader to possible underlying injury on these screening radiographs. While uncommonly obtained and inconsistently reported among the included studies, these comparison radiographs of the contralateral limb can enhance diagnostic confidence and accuracy [7]. The infrequent lateral and absent anterior displacement across the fracture may be secondary to the injury mechanism and/or regional myotendinous anatomy. Elbow immobilization was the most common method for treatment, and during follow-up, radiographs were frequently used to complement the clinical assessment and to detect residual findings, which occurred in almost half of the injured elbows. This latter finding emphasizes the need to reduce delayed diagnoses and risk for secondary injuries.

The current study has several limitations. First, the inclusion of studies over the past 60 years introduced heterogeneity in terms of available imaging modalities used for diagnosis, which is further compounded by the inclusion of case reports and case series that may highlight the use of a particular emerging imaging modality. For this reason, the current study examined not only the frequency of reported suspicion and/or visualization of each imaging modality, but also the diagnostic imaging modality that prompted treatment for each injured elbow. Second, the incomplete reporting of various findings and diagnoses across studies hindered additional subgroup analyses and led to the use of different denominators with different findings. Third, not all distal humerus physeal fractures were published, contributing to selection bias, and only those clinically and/or surgically-confirmed fractures were included, resulting in verification bias [37]. Finally, heterogeneity in the treatment approach and follow-up varied across studies, which hindered additional subgroup analyses to identify factors that associated with worse outcomes.

## Conclusion

This study showed that, while radiographs had overall low frequency of reported suspicious for physeal fractures, these were unanimously obtained as the first-line imaging modality in all injured elbows, which highlights the importance of recognizing subtle findings when interpreting these from extremely immature elbows of infants and toddlers. Forearm malalignment on these screening radiographs should lower the threshold for additional imaging, which can include radiographs of the asymptomatic contralateral elbow, ipsilateral ultrasound, and/or MRI of the symptomatic elbow. Future prospective studies are necessary to further define and optimize imaging workup and modality-specific protocols with the goal of reducing diagnosis delays and improving patient outcomes.

## Supplementary Information

Below is the link to the electronic supplementary material.

Supplementary file1 (DOCX 52 KB)

Supplementary file2 (DOCX 21 KB)

## Data Availability

Data is available upon reasonable request.
