# Supplementary material for: Distal humerus physeal fractures in children under 3 years: a systematic review and quantitative descriptive analysis
Source: Skeletal Radiol. 2026 Apr 21;55(9):2219–33. doi: 10.1007/s00256-026-05213-3 (PMC13369683; doi:10.1007/s00256-026-05213-3)
Supplement: Supplementary file 1 — Supplementary file1 (DOCX 52 KB) [file 256_2026_5213_MOESM1_ESM.docx]

**Supplemental – Database Search Strategy**

**PUBMED**

(("Humeral Fractures, Distal"[Mesh] OR "Humerus"[Mesh] OR "distal humerus"[tw] OR "distal humeral"[tw]) AND ("Epiphyses"[Mesh] OR "Growth Plate"[Mesh] OR "Salter-Harris Fractures"[Mesh] OR "physeal"[tw] OR "epiphyseal"[tw] OR "transphyseal"[tw] OR "epiphysis"[tw] OR "physis"[tw] OR "growth plate"[tw] OR "Salter-Harris"[tw]) AND ("fracture*"[tw] OR "separation*"[tw] OR "fracture-separation"[tw]) AND ("Infant"[Mesh] OR "Child, Preschool"[Mesh] OR "infant*"[tw] OR "neonate*"[tw] OR "neonatal"[tw] OR "newborn*"[tw] OR "toddler*"[tw] OR "pediatric"[tw] OR "paediatric"[tw] OR "child"[tw] OR "children"[tw])) AND (("Radiography"[Mesh] OR "radiograph*"[tw] OR "x-ray*"[tw] OR "xray*"[tw] OR "plain film*"[tw] OR "roentgenogra*"[tw]) OR ("Ultrasonography"[Mesh] OR "ultrasound"[tw] OR "ultrasonogra*"[tw] OR "sonogra*"[tw] OR "echogra*"[tw] OR "US"[tw]) OR ("Magnetic Resonance Imaging"[Mesh] OR "magnetic resonance imag*"[tw] OR "MRI"[tw] OR "MR imaging"[tw]))

**SCOPUS**

TITLE-ABS-KEY("distal humerus" OR "distal humeral") AND TITLE-ABS-KEY("physeal" OR "epiphyseal" OR "transphyseal" OR "epiphysis" OR "physis" OR "growth plate" OR "Salter-Harris") AND TITLE-ABS-KEY("fracture*" OR "separation*" OR "fracture-separation") AND TITLE-ABS-KEY("infant*" OR "neonate*" OR "neonatal" OR "newborn*" OR "toddler*" OR "pediatric" OR "paediatric" OR "child" OR "children") AND (TITLE-ABS-KEY("radiograph*" OR "x-ray*" OR "xray*" OR "plain film*" OR "roentgenogra*") OR TITLE-ABS-KEY("ultrasound" OR "ultrasonogra*" OR "sonogra*" OR "echogra*") OR TITLE-ABS-KEY("magnetic resonance imag*" OR "MRI" OR "MR imaging"))

**EMBASE**

('humerus fracture'/exp OR 'distal humerus' OR 'distal humeral') AND ('epiphysis'/exp OR 'growth plate'/exp OR 'Salter Harris fracture'/exp OR 'physeal' OR 'epiphyseal' OR 'transphyseal' OR 'epiphysis' OR 'physis' OR 'growth plate' OR 'Salter-Harris') AND ('fracture*' OR 'separation*' OR 'fracture-separation') AND ('infant'/exp OR 'preschool child'/exp OR 'newborn'/exp OR 'infant*' OR 'neonate*' OR 'neonatal' OR 'newborn*' OR 'toddler*' OR 'pediatric' OR 'paediatric' OR 'child' OR 'children') AND ('radiography'/exp OR 'radiograph*' OR 'x-ray*' OR 'xray*' OR 'plain film*' OR 'roentgenogra*' OR 'echography'/exp OR 'ultrasound' OR 'ultrasonogra*' OR 'sonogra*' OR 'echogra*' OR 'nuclear magnetic resonance imaging'/exp OR 'magnetic resonance imag*' OR 'MRI' OR 'MR imaging')

Additional filters were used to exclude book chapters, conference abstracts, expert opinions, meta-analyses, non-English articles, reviews, and systematic reviews.

**Supplemental Table 1. Modified Joanna Briggs Institute** **Criteria for Cases Reports (1 Patient per Article)**

| **Article** | **Q1^a^** | **Q2** | **Q3** | **Q4** | **Q5** | **Q6** | **Q7** | **Score (%)** | **Risk** |
| --- | --- | --- | --- | --- | --- | --- | --- | --- | --- |
| Arrigoni, Sini & Origo, 2022 [1] | Yes | Yes | Yes | Yes | Yes | Yes | Yes | 100 (7/7) | Low |
| Beckmann & Crawford, 2017 [2] | Yes | Yes | Yes | Yes | Yes | Yes | Yes | 100 (7/7) | Low |
| Brown & Eustace, 1997 [3] | Yes | Yes | Yes | Yes | Yes | Yes | Yes | 100 (7/7) | Low |
| Chand, 1974 [4] | Yes | Yes | Yes | Yes | Yes | Yes | Yes | 100 (7/7) | Low |
| Cohen *et al.*, 2006 [5] | Yes | Yes | Yes | Yes | Yes | Yes | Yes | 100 (7/7) | Low |
| Costa *et al.*, 2001 [6] | Yes | Yes | Yes | Yes | Yes | Yes | Yes | 100 (7/7) | Low |
| Dias, Lamont, & Jones, 1988 [7] | Yes | Yes | Yes | Yes | Yes | Yes | Yes | 100 (7/7) | Low |
| Downs & Wirth, 1982 [8] | No | Yes | Yes | Yes | Yes | Yes | Yes | 85.7 (6/7) | Low |
| Hansen et al., 2008 [9] | Yes | Yes | Yes | Yes | Yes | Yes | Yes | 100 (7/7) | Low |
| Hansen, Barnes & Tullos, 1982 [10] | Yes | Yes | Yes | Yes | Yes | Yes | Yes | 100 (7/7) | Low |
| Kamaci, Danisman & Marangoz, 2014 [11] | Yes | Yes | Yes | Yes | Yes | Yes | Yes | 100 (7/7) | Low |
| Lin, Liu & Zhang, 2016 [12] | Yes | Yes | Yes | Yes | Yes | Yes | Yes | 100 (7/7) | Low |
| Malik *et al.*, 2015 [13] | Yes | Yes | Yes | Yes | Yes | Yes | Yes | 100 (7/7) | Low |
| Merten, Kirks, & Ruderman, 1981 [14] | Yes | Yes | Yes | Yes | No | Yes | Yes | 85.7 (6/7) | Low |
| Moucha & Mason, 2003 [15] | Yes | Yes | Yes | Yes | Yes | Yes | Yes | 100 (7/7) | Low |
| Navallas *et al.*, 2013 [16] | Yes | Yes | Yes | Yes | Yes | Yes | Yes | 100 (7/7) | Low |
| Rose, Dixon & Dullon, 2002 [17] | Yes | Yes | Yes | Yes | Yes | Yes | Yes | 100 (7/7) | Low |
| Sawant *et al.*, 2002 [18] | No | Yes | Yes | Yes | Yes | Yes | Yes | 85.7 (6/7) | Low |
| Siddiqui [19]*et al.*, 2023 | Yes | Yes | Yes | Yes | No | Yes | Yes | 85.7 (6/7) | Low |
| Soyuncu *et al.*, 2009 [20] | No | Yes | Yes | Yes | Yes | Yes | Yes | 100 (7/7) | Low |
| Tharakan *et al.*, 2016 [21] | Yes | Yes | Yes | Yes | Yes | Yes | Yes | 100 (7/7) | Low |
| Triplet, Samora & Samora, 2018 [22] | Yes | Yes | Yes | Yes | Yes | Yes | Yes | 100 (7/7) | Low |
| Varghese *et al.*, 2017 [23] | Yes | Yes | Yes | Yes | Yes | Yes | Yes | 100 (7/7) | Low |
| Wang *et al.*, 2009 [24] | Yes | Yes | Yes | Yes | No | Yes | Yes | 85.7 (6/7) | Low |
| Arrigoni, Sini & Origo, 2022 [1] | Yes | Yes | Yes | Yes | Yes | Yes | Yes | 100 (7/7) | Low |

The Joanna Briggs Institute criteria for case reports [25] included seven domains: clarity of demographic information (Q1); a clearly outlined, chronological clinical history (Q2); an adequate description of the patient’s condition at presentation (Q3); a transparent account of diagnostic tests, assessment methods, and corresponding results (Q4); a clear description of the post-diagnosis clinical course (Q5); identification and discussion of any adverse or unanticipated events (Q6); and the inclusion of explicit takeaway lessons (Q7).

Articles that scored >70% were judged as low, between 50–69% as moderate, and < 50% as high risk of bias.

**Supplemental Table 2. Modified Joanna Briggs Institute Criteria for Case Series (≥2 Patients per Article)**

| **Article** | **Q1** | **Q2** | **Q3** | **Q4** | **Q5** | **Q6** | **Q7** | **Q8** | **Score (%)** | **Risk** |
| --- | --- | --- | --- | --- | --- | --- | --- | --- | --- | --- |
| Davidson *et al.*, 1994 [26] | Yes | Yes | Yes | No | No | Yes | Yes | Yes | 75.0 (6/8) | Low |
| Fette & Mayr, 2012 [27] | Yes | Yes | Yes | No | No | Yes | Yes | Yes | 75.0 (6/8) | Low |
| Galeotti *et al.*, 2023 [28] | Yes | Yes | Yes | Yes | Yes | Yes | Yes | Yes | 100 (8/8) | Low |
| Gigante *et al.*, 2017 [29] | Yes | Yes | Yes | No | No | Yes | Yes | Yes | 75.0 (6/8) | Low |
| Gilbert & Conklin, 2007 [30] | Yes | Yes | Yes | Yes | Yes | No | No | Yes | 75.0 (6/8) | Low |
| Jacobsen, Hansson & Nathorst-Westfelt, 2009 [31] | Yes | Yes | Yes | No | No | Yes | Yes | Yes | 75.0 (6/8) | Low |
| Kay *et al.*, 2017 [32] | Yes | Yes | Yes | No | No | No | No | Yes | 50.0 (4/8) | Moderate |
| Nimkin *et al.*, 1995 [33] | Yes | Yes | Yes | No | No | Yes | Yes | No | 62.5 (5/8) | Moderate |
| Oh, Park & Kyung, 2000 [34] | Yes | Yes | Yes | Yes | Yes | Yes | Yes | Yes | 100 (8/8) | Low |
| Paige & Port, 1985 [35] | Yes | Yes | Yes | No | No | Yes | Yes | Yes | 75.0 (6/8) | Low |
| Rogers & Rockwood, 1973 [36] | Yes | Yes | Yes | No | No | Yes | Yes | Yes | 75.0 (6/8) | Low |
| Siffert, 1963 [37] | Yes | Yes | Yes | No | No | No | Yes | Yes | 62.5 (5/8) | Moderate |
| Supakul *et al.*, 2015 [38] | Yes | Yes | Yes | Yes | Yes | Yes | Yes | Yes | 100 (8/8) | Low |

The Joanna Briggs Institute criteria for case series [39] included eight domains: presence of clear inclusion criteria for participants (Q1); standardized and reliable measurement of the condition for all participants (Q2); use of valid methods to identify the condition (Q3); consecutive inclusion of eligible participants (Q4); complete inclusion of all eligible participants within the specified timeframe (Q5); clear reporting of participant demographics (Q6); clear reporting of clinical information for each participant (Q7); and clear reporting of outcomes or follow-up results (Q8).

Articles that scored >70% were judged as low, between 50–69% as moderate, and < 50% as high risk of bias.

**REFERENCES**

1. Arrigoni C, Sini D, Origo C. Use of arthrography in managing neonatal epiphyseal distal humerus separation. *Minerva Orthop* 2022; 73

2. Beckmann NM, Crawford L. Salter-Harris I fracture of the distal humerus in a neonate: imaging appearance of radiographs, ultrasound, and arthrography. *Radiology case reports* 2017; 12:571-576

3. Brown J, Eustace S. Neonatal transphyseal supracondylar fracture detected by ultrasound. *Pediatric emergency care* 1997; 13:410-412

4. Chand K. Epiphyseal separation of distal humeral epiphysis in an infant. A case report and review of literature. *The Journal of Trauma: Injury, Infection, and Critical Care* 1974; 14:521-526

5. Cohen E, Rath E, Galil A, Atar D. Physeal separation of the distal humerus, a rare consequence of trauma at birth. case report. *Aktuelle Traumatol* 2006; 36:183-186

6. Costa M, Owen-Johnstone S, Tucker JK, Marshall T. The value of MRI in the assessment of an elbow injury in a neonate. *The Journal of bone and joint surgery British volume* 2001; 83:544-546

7. Dias JJ, Lamont AC, Jones JM. Ultrasonic diagnosis of neonatal separation of the distal humeral epiphysis. *The Journal of bone and joint surgery British volume* 1988; 70:825-828

8. Downs DM, Wirth CR. Fracture of the distal humeral chondroepiphysis in the neonate. A case report. *Clinical orthopaedics and related research* 1982:155-158

9. Hansen M, Weltzien A, Blum J, Botterill NJ, Rommens PM. Complete distal humeral epiphyseal separation indicating a battered child syndrome: a case report. *Archives of orthopaedic and trauma surgery* 2008; 128:967-972

10. Hansen PE, Barnes DA, Tullos HS. Arthrographic diagnosis of an injury pattern in the distal humerus of an infant. *Journal of pediatric orthopedics* 1982; 2:569-572

11. Kamaci S, Danisman M, Marangoz S. Neonatal physeal separation of distal humerus during cesarean section. *Am J Orthop* 2014; 43:E279-281

12. Rui-Lan L, Zhen-Jiang L, Li-Jun Z. Neonatal distal humeral physeal separation during caesarean section: a case report and review of a literature. *Int J Clin Exp Med* 2016; 9

13. Malik S, Khopkar SR, Korday CS, Jadhav SS, Bhaskar AR. Transphyseal injury of distal humerus: A commonly missed diagnosis in neonates. *J Clin Diagn Res* 2015; 9:SD01-02

14. Merten DF, Kirks DR, Ruderman RJ. Occult humeral epiphyseal fracture in battered infants. *Pediatric radiology* 1981; 10:151-154

15. Moucha CS, Mason DE. Distal humeral epiphyseal separation. *Am J Orthop* 2003; 32:497-500

16. Navallas M, Díaz-Ledo F, Ares J, et al. Distal humeral epiphysiolysis in the newborn: utility of sonography and differential diagnosis. *Clinical imaging* 2013; 37:180-184

17. Rose REC, Dixon R, Bullock R. Chondro-epiphyseal separation of the distal humerus in the newborn. A case report and review of the literature. *West Indian Med J* 2002; 51:268-271

18. Sawant MR, Narayanan S, O'Neill K, Hudson I. Distal humeral epiphysis fracture separation in neonates -- diagnosis using MRI scan. *Injury* 2002; 33:179-181

19. Siddiqui YS, Abbas MB, Anwer A, Abbas M, Chowdhry M, Khurana S. Bilateral Distal Humeral Physeal Separation-From Birth Trauma to Family Trauma. *Journal of orthopaedic case reports* 2023; 13:88-92

20. Söyüncü Y, Cevikol C, Söyüncü S, Yildirim A, Akyildiz F. Detection and treatment of traumatic separation of the distal humeral epiphysis in a neonate: a case report. *Ulus Travma Acil Cerrahi Derg* 2009; 15:99-102

21. Tharakan SJ, Lee RJ, White AM, Lawrence JTR. Distal humeral epiphyseal separation in a newborn. *Orthopedics* 2016; 39:e764-767

22. Triplet JJ, Samora WP, Balch Samora J. Distal humeral physeal separation in a newborn: a case report and review of the literature. *Curr Orthop Pract* 2018; 29:611-615

23. Varghese J, Teng M, Huang M, Balsam D. Birth injuries to growth plates: A sheep in wolves' clothing. *J Clin Ultrasound* 2017; 45:511-514

24. Wang P-H, Chern T-C, Su W-R, Jou IM. Ultrasonography applied in guiding the reduction and assessing the healing of distal humeral epiphysis fracture-separation in a neonate—A case report. *European Journal of Radiology Extra* 2009; 72:e91-e96

25. Moola S, Munn Z, Tufanaru C, et al. Chapter 7: systematic reviews of etiology and risk. In: Aromataris E, Munn Z, eds. *JBI manual for evidence synthesis*: JBI, 2020

26. Davidson RS, Markowitz RI, Dormans J, Drummond DS. Ultrasonographic evaluation of the elbow in infants and young children after suspected trauma. *The Journal of bone and joint surgery American volume* 1994; 76:1804-1813

27. Fette A, Mayr J. Slipped distal humerus epiphysis in tiny infants easily detected and followed-up by ultrasound. *Ultraschall Med* 2012; 33:E361-E363

28. Galeotti A, Zanardi A, Giacinto SD, Beltrami G, Cucca G, Lazzeri S. Transphyseal distal humeral separation in neonates: A case series. *Injury* 2023;

29. Gigante C, Kini SG, Origo C, Volpin A. Transphyseal separation of the distal humerus in newborns. *Chinese journal of traumatology = Zhonghua chuang shang za zhi / Chinese Medical Association* 2017; 20:183-186

30. Gilbert SR, Conklin MJ. Presentation of distal humerus physeal separation. *Pediatric emergency care* 2007; 23:816-819

31. Jacobsen S, Hansson G, Nathorst-Westfelt J. Traumatic separation of the distal epiphysis of the humerus sustained at birth. *The Journal of bone and joint surgery British volume* 2009; 91:797-802

32. Kay M, Simpkins C, Shipman P, Whitewood C. Diagnosing neonatal transphyseal fractures of the distal humerus. *Journal of medical imaging and radiation oncology* 2017; 61:494-499

33. Nimkin K, Kleinman PK, Teeger S, Spevak MR. Distal humeral physeal injuries in child abuse: MR imaging and ultrasonography findings. *Pediatric radiology* 1995; 25:562-565

34. Oh CW, Park BC, Ihn JC, Kyung HS. Fracture separation of the distal humeral epiphysis in children younger than three years old. *Journal of pediatric orthopedics* 2000; 20:173-176

35. Paige ML, Port RB. Separation of the distal humeral epiphysis in the neonate. A combined clinical and roentgenographic diagnosis. *Am J Dis Child* 1985; 139:1203-1205

36. Rogers LF, Rockwood CA, Jr. Separation of the entire distal humeral epiphysis. *Radiology* 1973; 106:393-400

37. Siffert RS. Displacement of the Distal Humeral Epiphysis in the Newborn Infant. *The Journal of Bone & Joint Surgery* 1963; 45:165-169

38. Supakul N, Hicks RA, Caltoum CB, Karmazyn B. Distal humeral epiphyseal separation in young children: an often-missed fracture-radiographic signs and ultrasound confirmatory diagnosis. *AJR American journal of roentgenology* 2015; 204:W192-198

39. Munn Z BT, Moola S, Tufanaru C, Tufanaru C, Stern C, McArthur A, et al. . Methodological quality of case series studies: an introduction to the JBI critical appraisal tool. *JBI Evidence Synthesis* 2020; 18:2127-2133
